# Basic emergency care course and longitudinal mentorship completed in a rural Neno District, Malawi: A feasibility, acceptability, and impact study

**DOI:** 10.1371/journal.pone.0280454

**Published:** 2023-02-06

**Authors:** Brown David Khongo, Kelly Schmiedeknecht, Moses Banda Aron, Prisca Nelisa Nyangulu, Wellington Mazengera, Enoch Ndarama, Andrea G. Tenner, Kimberly Baltzell, Emilia Connolly

**Affiliations:** 1 Partners In Health/Abwenzi Pa Za Umoyo, Neno, Malawi; 2 Department of Family Health Care Nursing, Institute of Global Health Sciences, University of California San Francisco, San Francisco, California, United States of America; 3 Ministry of Health, Neno, Malawi; 4 Department of Emergency Medicine WHO Collaborating Centre for Emergency, Critical, and Operative Care, University of California San Francisco, San Francisco, California, United States of America; 5 Division of Pediatrics, University of Cincinnati College of Medicine, Cincinnati, Ohio, United States of America; 6 Division of Hospital Medicine, Cincinnati Children’s Hospital, Cincinnati, Ohio, United States of America; Bay Area Hospital, North Bend Medical Center, UNITED STATES

## Abstract

**Background:**

Frontline providers mostly outside specific emergency areas deliver emergency care around the world, yet often they do not receive dedicated training in managing emergency conditions. When designed for low-resource settings, emergency care training has been shown to improve provider skills, facilitate efficient use of available resources, and reduce death and disability by ensuring timely access to life-saving care.

**Methods:**

The WHO/ICRC Basic Emergency Care (BEC) Course with follow up longitudinal mentorship for 6 months was implemented in rural Neno District Malawi from September 2019-April 2020. We completed a mixed-methods analysis of the course and mentorship included mentor and participant surveys and feedback, mentorship quantification, and participant examination results. Simple descriptive statistics and boxplot visuals were used to describe participant demographics and mentorship quantification with a Wilcoxon signed-rank test to evaluate pre- and post-test scores. Qualitative feedback from participants and mentors were inductively analyzed using Dedoose.

**Results:**

The median difference of BEC course examination percentage score between participants before the BEC course and immediately following the course was 18.0 (95% CI 14.0–22.0; p<0.001). Examination scores from the one-year post-test was lower but sustained above the pre-course test score with a median difference of 11.9 (95% CI 4.0–16.0; p<0.009). There were 174 mentorship activities with results suggesting that a higher number of mentorship touches and hours of mentor-mentee interactions may assist in sustained knowledge test scores. Reported strengths included course delivery approach leading to improved knowledge with mentorship enhancing skills, learning and improved confidence. Suggestions for improvement included more contextualized training and increased mentorship.

**Conclusion:**

The BEC course and subsequent longitudinal mentorship were feasible and acceptable to participants and mentors in the Malawian low resource context. Follow-up longitudinal mentorship was feasible and acceptable and is likely important to cementing the course concepts for long-term retention of knowledge and skills.

## Introduction

Basic emergency care is essential along with every level of the health care continuum from the community to tertiary care [1]. In Malawi, emergencies are seen along the care continuum without specified emergency units until district or tertiary level central hospitals. Frontline providers mostly outside specific emergency areas deliver emergency care every day around the world, yet often they do not receive dedicated training in managing emergency conditions. This is true in the rural district of Neno in southwestern Malawi, where there are very few practicing physicians and providers with prior emergency care training. Most patient care is completed by providers such as medical assistants, clinical officers, and nurses who lack routine emergency care training and supervision. Thus, the effectiveness of care delivered in limited-resource settings like Neno is often compromised by inadequate emergency supplies, lack of triage systems, failure to recognize and provide initial management for emergent conditions, and delayed referral to advanced care. All of these factors result in avoidable mortality and morbidity [1–5]. When designed for low-resource settings, emergency care training has been shown to improve provider skills, facilitate more efficient use of available resources, and reduce death and disability by ensuring timely access to even simple life-saving treatments [6–14]. However, access to context-appropriate emergency care education courses, high-quality trainers, simulation materials, and ongoing treatment support is limited.

Of the many existing emergency care courses available, most are limited in scope (such as focusing on only one aspect of emergency care such as trauma), access, or cost [15–23]. Many of these courses originate in high resource settings and include diagnostics or technology that are unavailable in most care settings globally with omitting considerations for low resource context. Frequently these courses require expensive materials and monitoring as well as sponsorship or linkage to existing infrastructure and training centers that are not possible for most hospitals and providers around the world [24]. Additionally, many of the existing emergency care education courses situate physicians at the helm of care delivery, failing to consider that most of the emergency care in places like Neno district is provided by nurses and other providers with fewer opportunities for specialization and training in emergency care.

In response to this paucity of context-specific and open-access emergency care training materials, the World Health Organization (WHO) with the International Committee of the Red Cross (ICRC) and the International Federation for Emergency Medicine (IFEM) created the course *Basic Emergency Care (BEC)*: *Approach to the acutely ill and injured*. This low-fidelity, open-access BEC course is designed for use in a broad range of providers with little to no prior emergency care experience or training and potentially limited resources with a systematic approach to early identification and timely care to save lives. The course covers a systematic "ABCDE" approach to examine and treat acutely ill patients who may not already have a differentiated diagnosis. The course then covers more in-depth conditions related to trauma, difficulty in breathing, shock, and altered mental status and has been successfully implemented in Tanzania, Uganda, and Zambia [25].

The BEC course was provided to providers at two hospitals in the rural Neno District in Malawi through the WHO Global Emergency and Trauma Care Initiative, in collaboration with the University of California San Francisco (UCSF) WHO Collaborating Center for Emergency, Critical, and Operative Care, Partners In Health/Abwenzi Pa Za Umoyo (PIH/APZU), and the Malawi Ministry of Health (MOH) in Neno District, and Malawi College of Medicine. The course was followed by six months of longitudinal mentorship from local clinical providers. We report here on feasibility, acceptability, and impact of the course and mentorship from the participants and mentors along with mentorship quantification with the pre-, post-, and one-year post-course performance on content-based examinations.

## Methods

### Study setting

**Fig 1:**
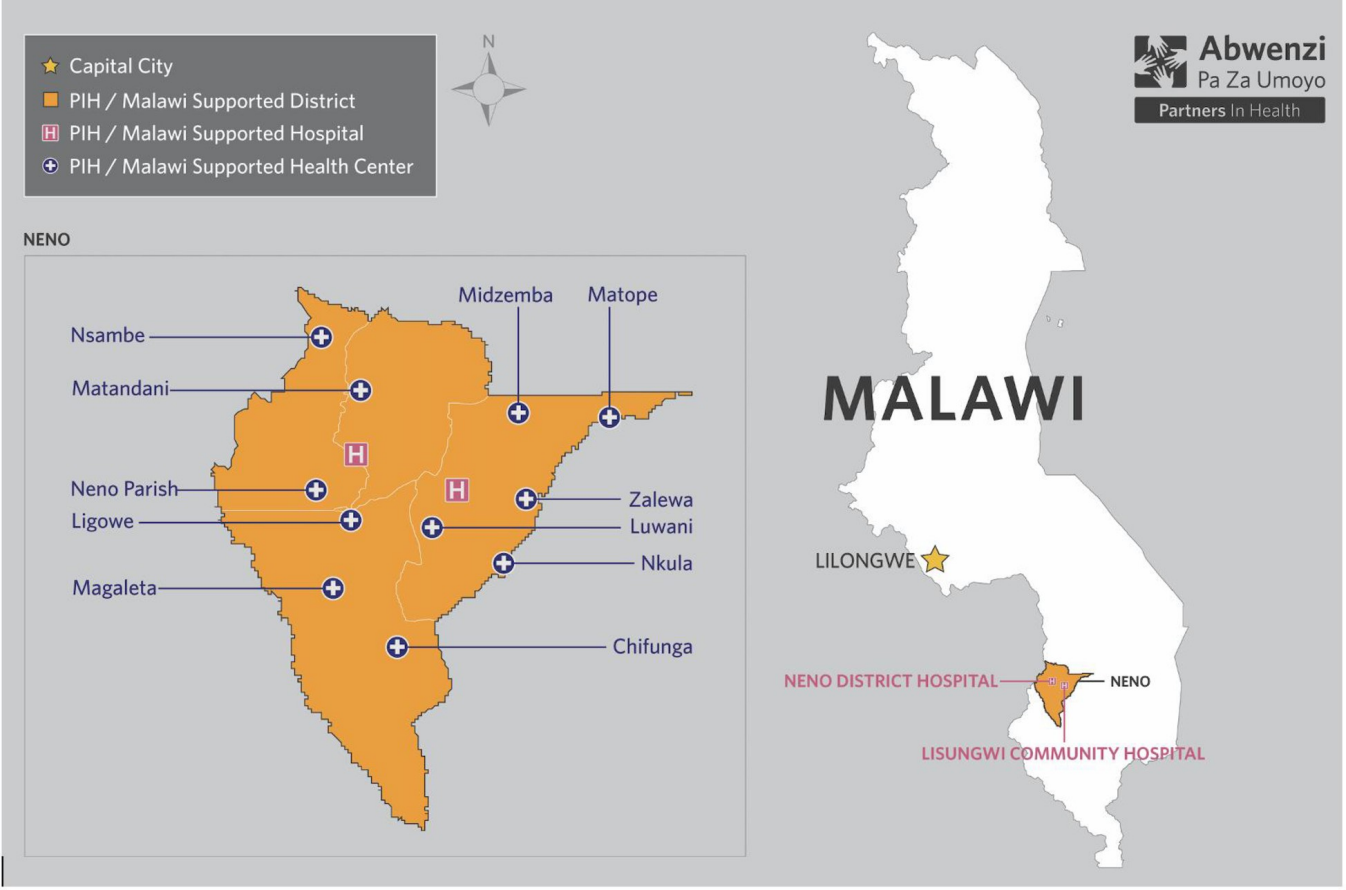
Map of Neno District, Malawi: Republished from partners In Health under a CC BY 4.0 license, with permission from Ermyas Birru, original copyright 2014.

Malawi is a low-income country in sub-Saharan Africa with approximately 18.9 million people projected in 2021 including approximately three million children under 5 years. Injuries and trauma account for an estimated 19% of all non-communicable diseases and injuries (NCDIs) disability-adjusted life years (DALYs) in Malawi and contribute to 6.4% of all deaths [26–28]. Neno is located in the southwest of Malawi and is one of the hardest to reach as the last district without a paved road to its district hospital. It is one of the most impoverished areas of the country with only 3.7% of the population having access to electricity and >70% living in poverty [29]. In the district, there are two secondary level care hospitals; Neno District Hospital (NDH) and Lisungwi Community Hospital (LCH) along with 13 primary health facilities and a robust community health worker program (Fig 1).

### Study design

This was a mixed-methods analysis that included mentor and participant surveys, mentorship quantification, and participant examination results. The mentor and participant surveys and participant examinations were completed before and after the BEC course and one year following the BEC course intervention. This study received ethical approval from the National Health Science Research Committee (NHSRC) in Malawi with protocol number 1059 titled “*Lessons Learned from Monitoring and Evaluation of Community Health Initiatives in Neno District*, *Malawi*." In addition, ethical approval was received from UCSF Committee for Human Research with protocol #19–28037 titled “*Piloting the WHO Basic Emergency Care Course and Trauma Checklist in Neno District*, *Malawi*.*”*

### BEC course and participants

Two BEC courses were completed from September to October 2019 at the two hospitals in the district, Neno District Hospital and Lisungwi Community Hospital, as all emergent or urgent patients in the district either seek direct care or are referred to these facilities for treatment. All course materials were delivered in English and formally hosted by local and National MOH and PIH/APZU.

Master trainers from UCSF and the Africa Federation for Emergency Medicine (AFEM) conducted a two-day training of trainers (ToT) on teaching methodology and delivery mechanisms for key BEC concepts, after which the newly trained facilitators and the master trainers taught the course to local participants. Two local district-based mentors were selected by hospital leadership based on leadership ability, level of commitment, and interest in clinical care to provide ongoing mentoring on BEC practices in the district. Neither one had prior formal emergency care training or primarily worked in acute care. The goal of the ToT was to develop BEC facilitators who could then train and mentor additional staff using the BEC curriculum.

Following the ToT, two BEC courses (one at each hospital) were taught over five days in September and October 2019 by two master BEC trainers and the newly-trained facilitators with supervision. Participants of mid-level providers and nurses were selected by hospital leadership with no prior formal emergency care training and never primarily worked in trauma and/or acutely ill patient settings, but all care for these patients as part of their daily job. The topics comprised didactic lectures, interactive workbook questions, case scenarios, and hands-on skills sessions. Each lecture had small group sessions where students practiced case scenarios and discussed how to manage acutely ill patients. Hands-on skills stations allowed participants to learn and practice skills using available supplies. Examples of hands-on skills taught in this course included: airway skills like inserting oropharyngeal airways and how to perform airway maneuvers to open an airway; breathing skills such as what oxygen delivery device to use, and emergency needle decompression; and circulation skills such as controlling bleeding and uterine massage. To complete the course, participants had to complete all activities in their workbooks, pass all skills tests through the performance of all critical actions, successfully outline the management of one case scenario, pass a post-test with a score of *>*75% and attend all sessions. Assessments included pre-and post-course content-based evaluations covering course contents, skills assessments, workbook completion checks, and a post-course survey. Written consent was obtained from each participant, trainer, and mentor after explanation from the facilitator prior to the course for use of generated data.

### Mentorship

The healthcare workers who completed the BEC course were given six months of formal follow-up mentorship completed by the two BEC facilitators, with one located at Neno District Hospital and Lisungwi Community Hospital respectively. Immediately following the BEC course completion in October 2019, on-site mentorship was initiated. Mentors were expected to be in the outpatient department or hospital wards mentoring for at least two to three days a week for 5 hours a day over six months. Additionally, mentors were available for staff to call when there were emergencies or acute care. The mentorship specifically included observation and assistance with emergency cases along with consultation, teaching, and meetings. Mentors’ activities included compiling and maintaining an emergency supply box that would be easily accessible to staff, assisting and educating both BEC trained and untrained staff during emergency cases, planning skills or case scenario teaching throughout the wards and outpatient departments, and participating in meetings surrounding emergency care in the district. They also collected data on their mentorship with monthly reporting for the six months following the training in the two hospitals using CommCare application. Data was reviewed with monthly check-ins with a BEC master trainer as a supportive supervisor for coaching and guidance as well as surveys on perceptions of the mentorship.

### Data collection

#### BEC course and mentorship quantitative evaluation

A pre-and post-test evaluation was collected for each participant immediately before and after the BEC course in September and October 2019 with an additional examination at one year following the BEC course in November 2020 [30].

Mentorship data was collected beginning in October 2019 through April 2020 on each mentorship or teaching session including the name of the mentor, time of mentorship, number of emergency patients seen in mentorship activities, the clinical area within the hospital (outpatient department (OPD) or inpatient wards (maternity, general adult or pediatric ward), type of mentorship activities (direct patient care, observation, equipment checklist, individual teaching or mentoring, meetings or review of a condition or skill with staff), the requirement for direct patient care (no assigned staff, staff not around, patient too sick for number of assigned staff, not enough staff for number of patients), skills used and taught, and equipment and medications used.

#### BEC course and mentorship qualitative survey evaluation

Post BEC course surveys in English were completed by participants immediately at the end of the BEC courses in September and October 2019. The post-course survey assessed course strengths and content, instructor evaluation if the course was appropriate to the setting, suggestions for improvement, mentorship need, and other comments. Additionally, programmatic evaluation surveys in English were evaluated with available participants in November and December 2020—one year following the BEC course. The programmatic evaluation survey assessed if the course improved knowledge and skills if the mentorship component improved understanding and retention of concepts with further learning, suggestions for improvement for the course and mentorship, and additional suggestions and comments for improvement of the BEC course and mentorship.

Mentorship surveys were conducted with the two mentors immediately at the end of the BEC courses in September and October 2019. Every month for six months during the supported period of mentorship, mentors had the opportunity to write in comments and descriptions during mentorship activities along with qualitative feedback on mentorship program strengths, challenges, and limitations of the mentorship program and the programmatic setting, suggestions for improvement and other comments.

### Data analysis

Data from pre, post-course, and one-year post-course examinations and survey data were analyzed in R version 4.0.2 [31] using Rstudio Integrated Development Environment Version 1.4.1103 [32]. A suite of “Tidyverse” packages was used to clean, manage, and analyze the data. Simple descriptive statistics and visuals (boxplot) were used to describe participant demographics and course scores. A Wilcoxon signed-rank test was used to evaluate the difference in the median between pre-and post-test scores for Lisungwi Community Hospital, Neno District Hospital, and combined. Simple counts were used to demonstrate mentorship characteristics and the total number of mentorship activities, hours of mentorship, and the number of emergency patients.

Responses from the pre, post-course and one-year post-course surveys as well as the mentor surveys and monthly qualitative feedback were uploaded into Dedoose version 8.3.17 for data management. The data was analyzed using qualitative content analysis by three authors with immersion and active reading of transcripts. To ensure the reliability of coding and consistency, three authors independently read the transcripts line by line to inductively assign codes to similar concepts that repeatedly emerged from the data in line with study objectives. The codebook was generated through iterative review with the final codebook agreed upon by joint consensus. We identified relationships between these codes, repeatedly identified codes were merged and themes around facilitators and challenges to execution of the basic emergency clinical care were generated. We chose quotes for each theme summarizing main points established from the qualitative data.

## Results

There were 32 participants in two BEC courses in Neno District. One course was completed at the Neno District Hospital with 18 participants and one in Lisungwi Community Hospital with 14 participants (Table 1). In both courses at Lisungwi Community Hospital and Neno District Hospital, there were more nurses (59.4%) compared to clinicians (40.6%) with more males than females (71.9% compared to 28.1%). Facilitators included emergency care specialists, nurses, and clinical officers.

**Table 1:** Participant characteristics.

	Neno District Hospital	Lisungwi Community Hospital	All participants
Total participants (*n)*	18	14	32
**Participant cadre**			
**Nurses *n(%)***	10 (55.6%)	9 (64.3%)	19 (59.4%)
Clinicians* *n(%)*	8 (44.4%)	5 (35.7%)	13 (40.6%)
**Sex**			
**Male *n(%)***	11 (61.1%)	12 (85.7%)	23 (71.9%)
**Female *n(%)***	7 (38.9%)	2 (14.3%)	9 (28.1%)

*Includes mid-year providers of clinical officers and medical assistants

**Fig 2:**
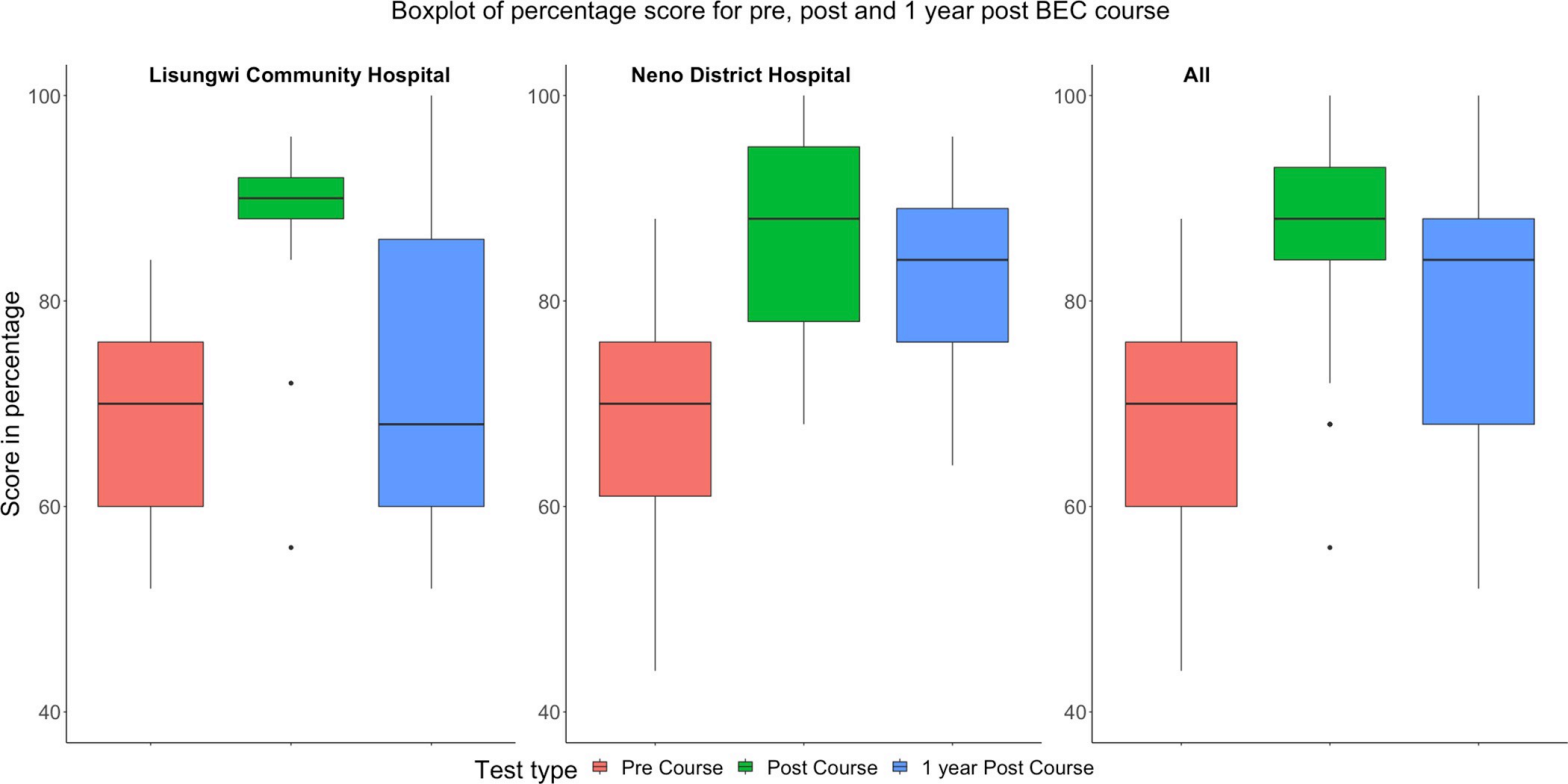
Box Plot of scores (in percentage) for pre-course, post-course and one-year post-course after the BEC course.

Knowledge tests were conducted before the BEC course (pre-course test), immediately following the BEC course (post-course test), and one year following the BEC course (one-year post-course test) (Table 2). Pre-course tests from Neno District Hospital averaged 69.8% correct and the Lisungwi Community Hospital was slightly lower at 68.3%. Post-course tests overall had improved knowledge of basic emergency care by mean test percentages. Lisungwi Community Hospital was slightly higher with an average of 87.1% correct and Neno District Hospital with an average of 85.8% correct immediately after the course completion. Overall the median difference of percentage score between participants before the BEC course and immediately following the course was 18.0 (95% CI 14.0–22.0; p<0.001). The knowledge for all participants from the one-year post-test was lower but sustained from the pre-course test with a median difference of 11.9 (95% CI 4.0–16.0; p<0.009). There was more variability in test scores for the one-year post-course test especially among participants from Lisungwi Community Hospital due to small sample sizes. The BEC pre- and post-tests are not disaggregated into specific clinical domains for a sub-analysis of specific knowledge areas. At the one-year post BEC course, there was 73% and 67% of staff retained for the assessment at Lisungwi Community Hospital and Neno District Hospital, respectively. Of the 10 participants lost (6 clinicians and 4 nurses), seven were due to transfers out of the district, one left the Ministry of Health for another organization and two were due to restructuring of Partners In Health. The box plot distribution of scores for pre, post, and one-year post-BEC courses with statistically significant changes are shown in Fig 2.

**Table 2:** Pre-course, post-course and one-year post-course mean test scores with median difference in Lisungwi Community Hospital and Neno District Hospital.

	Pre-course test	Post-course test	One-year post-course test	Median Difference with p-value^^^ between pre-course and post-course test (95% CI)	Median Difference with p-value^^^ between pre-course and 1 year post-course test (95% CI)
	Mean	Mean	Mean		
**Lisungwi Community Hospital** *	68.3	87.1	74.2	19.9 (13.9–24.0) P = 0.001	4.0 (8.0–20.0) P = 0.4749
**Neno District Hospital** **	69.8	85.8	83	18.0 (8.0–23.9) P = 0.002	12.0 (4.0–20.0) P = 0.0045
**All participants** ^#^	69.1	86.4	78.8	18.0 (14.0–22.0) P<0.001	11.9 (3.9–16.0) P = 0.009

*Sample sizes for Lisungwi Community Hospital (LCH) are 15 for pre- and post-course test and 11 for one-year post-course test

**Sample sizes for Neno District Hospital (NDH) are 18 for pre- and post-course test and 12 for one-year post-course test

^#^Sample sizes for all participants are 32 for pre- and post-course test and 23 for one-year post-course test

^^^Wilcoxon t- test comparing median difference between tests

Mentorship characteristics were broken down into the number of mentorship activities per clinical area, type of mentorship activities, and reasons for mentors being required for direct patient care during a mentorship session (Table 3). Overall there were 402 mentorship activities with most being completed in the outpatient department where emergency patients are first received upon arrival to the hospital. Observation of other providers with coaching and mentorship was the predominant mentorship activity with 61 instances of direct patient care by the mentors. Within these instances of direct patient care, the most common reason for hands-on mentorship involvement was due to the patient being too sick for the number of assigned staff. However, there were 24 instances where there was no staff assigned or not enough staff for the number of patients.

**Table 3:** Mentorship characteristics.

	Neno District Hospital	Lisungwi Community Hospital	Total
**Number of Mentorship Activities per Clinical Area** *			
Outpatient Department	64	81	145
Adult Inpatient Ward	76	54	130
Pediatric Inpatient Ward	21	68	89
Maternity Ward	21	13	34
Other	1	3	4
**Type of Mentorship Activities** *			
Direct patient care	30	31	61
Observation of other providers	96	68	164
Equipment checklist	12	20	32
Individual teaching or mentoring	16	37	53
Meetings	18	5	23
Review a condition or skill with staff	4	7	11
**Reasons Mentor was Required for Direct Patient Care** *			
No staff assigned	10	3	13
Assigned staff not around	3	4	7
Patient too sick for number of assigned staff	13	14	27
Not enough staff for number of patients	1	10	11
Other/blank	3	2	5

*Mentors could choose more than one clinical area, type of mentorship and requirement for direct patient care.

Overall mentorship at Neno District Hospital had more mean mentorship activities than Lisungwi Community Hospital (16.3 per month versus 12.7 per month), with more mentorship hours (76.7 per month versus 68.8 per month) and more emergency patients seen in mentorship activities (31.7 per month versus 17.2 per month) (Table 4). When comparing quantitative evaluations, BEC participants at Neno District Hospital maintained their knowledge, on average, over the year with six months of mentorship, while participant scores at Lisungwi Community Hospital decreased by 15%.

**Table 4:** Total number of mentorship activities, hours of mentorship and number of emergency patients.

	Neno District Hospital	Lisungwi Community Hospital	Total
**Number of Mentorship Activities** Total n (mean per month)	98 (16.3 per month)	76 (12.7 per month)	174 (14.5 per month)
**Number of Mentorship Hours** Total n (mean per month)	460 (76.7 per month)	383 (68.8 per month)	843 (70.3 per month)
Number of Emergency Patients Seen in Mentorship Activities* Total n (mean per month)	190 (31.7 per month)	103 (17.2 per month)	293 (24.4 per month)

*Subset of total patients seen in mentorship activities

### Participant and mentor survey results

Following the course, all the participants who responded to the post-course survey (N = 27) agreed the course content was “very appropriate”, and 89% (N = 24/27) felt that the course content was “very appropriate” to the setting and context. All of the respondents to the course survey either “agreed” or “strongly agreed” that the BEC was a productive use of their time and would recommend the course to other healthcare providers. When asked what they would change about the course, just under half of the participants felt the course should be longer or follow up with refresher training.

### Participant and mentor qualitative feedback

**Fig 3:**
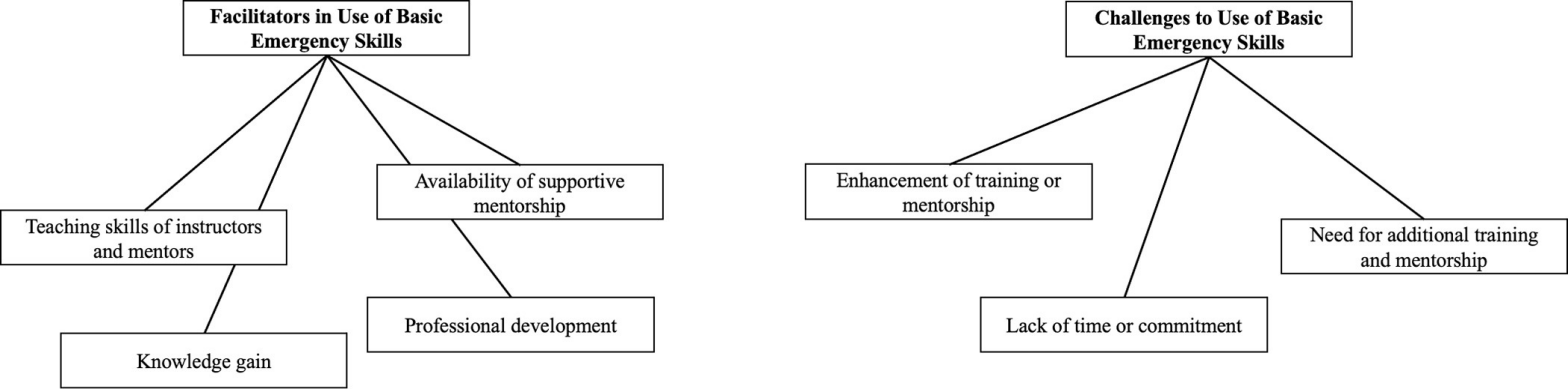
Mapping themes of facilitators and challenges of basic emergency skill use in Neno District.

Through examination of the survey responses and feedback, findings were deductively identified around facilitators and challenges in use of basic emergency care enabled by the BEC training and mentorship (Fig 3). Themes of facilitators included i) teaching skills of instructors and mentors, ii) availability of supportive mentorship; iii) knowledge gain, and iv) professional development. The themes of challenges were i) the need for additional training or mentorship, ii) enhancement of training or mentorship; and iii) lack of time or commitment to the use of basic emergency skills.

### Facilitators in use of basic emergency skills

The teaching and inviting nature of the BEC instructors and mentors, along with hands on skills sessions were key enablers to use of basic emergency skills and comfort in use for participants. Participants shared that the instructor encouragement and ability to approach them were vital for engagement and learning. Participants remarked “[They were] clear, and skilled with making everyone comfortable,” “They were flexible which made us comfortable to ask questions and clarifications” and “They were good, very clear on mentioning things to be done, very friendly and loving. Could answer any questions we asked, and we love that.” The instructors also enhanced confidence in skills through teaching. One participant responded that the course “Necessitated the acquisition of the necessary skills, confidence in handling emergencies and improved morale” and another noted “[Instructors] involved participants to enhance participation.” Use of hands-on skills were valued as well with one participant stating, “Knowing how to manage trauma and cervical spine immobilization were possible with skills sessions.”

Increased knowledge and skills gained was a fundamental enabler for participants to use basic emergency skills for their patients. One participant remarked that “knowledge gained during the BEC course is being applied on my patients with good outcomes” and another responded, “I managed and helped poly-trauma patient from a road trauma accident using the knowledge and skills gained.” Due to the training and mentorship, participants felt confidence and pride in patient management. One respondent offered “before this course I used to run away from emergency patients but now I am able to manage them.”

The availability of supportive mentorship following the BEC course was an enabler for continued learning and correct use of basic emergency skills. Reminding participants of key concepts was a key theme with one respondent offering “[the] mentor helped with clarifying the concepts I missed during the training” and another remarked “[Mentors] helped with sharpening skills attained and created more time for practice”. From the mentorship, improved quality of care was noted–“[Mentors] improved quality of care through presence and reminders,” “Mentorship assisted in my weak areas,” and “Mentor was always present when needed during emergencies. Mentors remind when one forgets.”

Over the six months of mentorship, the mentors also shared that their work improved knowledge and skills in the participants. One mentor stated, “The mentorship program is of paramount importance in our field…In my setting, it [mentorship] was particularly beneficial to those who were new and not trained as they were eager to be equipped with knowledge and skills.” Both mentors shared that they observed improvements in participants. One shared, “[The] provider was able to adjust management quickly as the child was deteriorating very fast and managed to save him,” and another stated, “[The] mentee used BEC skills to save life by calculating and giving appropriate amount of fluids to a baby in shock.”

Furthermore, and most important, the mentors gave feedback frequently on how longitudinal hands-on mentorship allowed them to constructively correct practice of mentees to improve use of basic emergency skills and quality of care delivered to patients. One mentor gave an example, “provider forgot to check pulse rate, sensation and capillary refill. She was mentored and reminded how to do splints. Finally, she checked everything correctly,” and another mentor noted “provider was able to resuscitate baby successfully after being reminded of the right way to provide bag mask valve [resuscitation] that was age appropriate, commenced baby on oxygen therapy then sent them for proper management.”

Lastly, the instruction and mentorship of the BEC course allowed mentors and participants to build their professional attributes. One mentor shared “I would say I feel great and motivated to have participated in [the] BEC and mostly to be a mentor of the project. Personally, the trainings and mentorships were great. They have improved my skills, knowledge and my CV.” A participant noted the BEC training and mentorship “updated/refreshed my attitude, knowledge and skills for emergency care.”

### Challenges to use basic emergency skills

A challenge that was voiced clearly by the participants was the need for additional and refresher training, and continued mentorship. One participant remarked “duration of course was too short with a lot of content to cover” and another offered “would suggest a refresher course and course review meetings”. In appreciation of the knowledge gained during the training, all participants felt that all healthcare providers could benefit from the BEC course and mentorship. Several respondents recommended increasing the number of participants in the course; “train everyone at the facility so cases always find trained personnel.” Overwhelmingly the participants recommended more mentorship availability. One stated “more time needed with mentor especially at night” and another asked that “[the] mentor should have a schedule to follow up on all mentees”. One participant even suggested avoiding overwhelming mentors with the requests “do more group mentorship if time constraints.”

In addition to extended training and mentorship, there were participant and mentor calls for enhancement of the training and mentorship. With acknowledgment of the knowledge and skills the BEC program had provided. Suggestions for enhancement of the training focused on further resources and more skills training, for example “Need more case scenarios as their use bring an idea of how to approach different patients with different presentations,” and “make some resources available like mannequins for continued and updating skills.” One adjustment that was noted by several participants was for the master trainers to be Malawian or aware of local guidelines with statements including, “there was a need to have some local facilitators to give local scenarios” and “lecturers to be aware of national guidelines so as to be able to deliver real situations and management according to the setting.” Participants did not have specific areas for refreshing training but rather several noted it was needed to maintain knowledge and update participants to current recommendations.

For enhanced mentorship there were calls for more interface and additional mentors with participants requesting, “Add drills for one-on-one mentorship for the team” and “more mentors at departmental level would make the program better.” Some participants expressed a desire for additional structure to the mentorship with noting, “Mentors should have a schedule to cover/follow up on all mentees, “and “[there is] need to draw action plans with mentors to assess progress over time.”

Even though the mentorship was appreciated by both mentors and mentees with numerous examples of improved patient care, challenges for the mentors included the time mentorship took that was in addition to their other duties. One stated, “the time given to us to work is unrealistic and it was not working. We were still being called to attend emergencies day and night even if we were not supposed to handle emergencies on that particular day or when we were off duty.” Additionally, the limitation of staff shortage and loss of trained providers limited the reach of the mentors. One noted “shortage of staff doesn’t help much either because only a few were trained; we have one person on duty most of the time giving less time for mentorship as they are always busy with other cases that may not be emergencies.” In addition to staff shortage, a limitation noted was lack of motivation by mentees with one mentor remarking “there is need for motivation and mentoring of nurses to do the right things at the right time.” Another mentor suggested that the participants for the BEC course should be carefully chosen so that they are assigned to the outpatient department and wards where BEC skills are utilized and plan on remaining working in the Ministry of Health in the district. The mentor explained, “…[There was a] lack of availability and commitment of trained providers. This happen because inappropriate people were trained and they were mostly busy attending their usual duties neglecting BEC.”

## Discussion

The BEC course and follow-up mentorship were well received by participants and mentors in the Neno district with positive feedback on the acceptability of the course content and mentorship in the context, with suggestions to enhance support and use of basic emergency skills. The quantitative and qualitative results from this project demonstrate the feasibility and impact of implementing the BEC course with longitudinal mentorship by peer providers in resource-limited settings like rural Neno, Malawi.

Following the BEC course, there was a statistically significant improvement for all participants in knowledge on the immediate post-course exams. The improvement on the immediate post-course exam demonstrated that the course materials and training were sufficient and feasible for significant knowledge acquisition. Qualitatively, participants and mentors agreed that the BEC course content was a productive use of their time, had valuable learning in caring for patients with emergencies, and was appropriate to the context of a rural district hospital. Speaking to the acceptability of the course, participants and mentors encouraged further sessions of the course through refresher training and course review meetings as well as recommendations to include all health care workers in the district with the priority of training those with direct responsibility for acute care patients. A recommendation that is key in planning further BEC courses and mentorship programs was the need to contextualize and adjust curriculum and skills sessions to the local country or even district context to best aid the participants in hands-on practice.

These results show that it is possible to increase knowledge using the context-relevant BEC course in the identification and management of emergency conditions while not depending on highly technical diagnostic or treatment interventions. Our quantitative results agree with and add to prior findings of the BEC course pilot evaluating feasibility, appropriateness, and potential challenges of the training in Tanzania, Uganda, and Zambia [6]]. The pilot evaluation indicated that there was successful knowledge transfer in the written and skills-based evaluation and the course was well received by facilitators and participants who deemed the information context-appropriate. Tanzania, Uganda, and Zambia pilot course results recommended further studies since no long-term knowledge assessment or impact on clinical practice were done following the course. However, our study adds assessment of knowledge and acceptability with hands-on longitudinal mentorship following the BEC course.

In this study, there was long-term retention of knowledge at one year following the BEC course in the context of six months of mentorship. Knowledge was slightly lower but sustained from the pre-course exam with statistically significant knowledge retention overall. Participant knowledge retention was higher at Neno District Hospital overall than Lisungwi Community Hospital one year following the BEC course and had higher mentorship hours for six months, suggesting that additional mentorship can improve retention of knowledge. Due to the lack of a control group and the overall small sample size, we cannot confirm causation of sustained knowledge was mentorship but it is not unexpected that the mentorship aided in maintaining knowledge and practice for participants.

Qualitatively, participants reported that mentorship was vital for correcting clinical practice, reinforced concepts of BEC and enhanced skill building with improved patient care that, in turn, increased participants’ confidence and pride in emergency patient care. Furthermore, the participants suggested having more structured and interactive time with mentors in future interventions which can be seen in the quantitative results as well with the lack of time for reinforcing clinical skills and reviews of conditions. From the perspective of the mentors, they felt that the course and mentorship program was beneficial to their knowledge and skills gained as well as professional growth. Challenges in mentorship were balancing additional mentorship time with regular clinical duties and lack of mentee involvement with staff turnover after BEC training, poor commitment in some cases to skill building, and small numbers of staff.

Despite numerous short-term training and educational opportunities, many healthcare providers continue to display an inadequate performance to provide timely, high-quality care [33, 34]. Several systematic reviews have been conducted for strategies to improve performance and hence patient outcomes [35–42]. Utilizing a single analytical approach in a systematic review [41] and in the secondary analysis [42], research shows that training alone only has a moderate impact on effect size compared to on-site training and mentorship, with increased knowledge by 18–18.8% points. Similarly, on-site mentorship has shown to improve clinical competencies and performance and is well recognized in medicine [43–45]. However, relatively little has been written about mentoring in emergency medicine or with the BEC course with the need for further investigation in different contexts and settings [45, 46]. This study has demonstrated that longitudinal mentorship following training may sustain knowledge acquired during a short one-week BEC course with reported high acceptability of the programme with mentorship by participants. Overall, there were 174 mentorship activities over six months with 293 patients seen with mentors over 843 total hours. These findings demonstrate a significant amount of time and reinforcement of BEC skills was possible through mentorship that assisted in correction and improvement in skills among mentees. The results further suggest that a higher number of mentorship touches and hours of mentor-mentee interactions may assist in sustained knowledge test scores on the one-year post-course test with more direct comparisons required in further evaluations. The authors plan to continue provision of BEC courses for which a second one was completed in November 2021, refresher trainings and mentorship in Neno District with interest on the impact on participant knowledge and skill attainment as well as morbidity and mortality trends in comparison to the pre-implementation of BEC course and mentorship.

### Limitations

This study is limited in generalizability by its geographical small footprint in one district in Malawi and a small number of participants practicing only in hospitals along with staff turnover during the course of the study. However, these challenges are not unlike other rural settings across sub-Saharan Africa and Asia representing the environment of work. The other limitation is that the study could not follow patient level outcomes since it would be difficult to attribute the outcomes to the course and mentorship alone. The future studies should be designed to follow the effect of the BEC training and mentorship on patient level outcomes as well as sub-analysis by health worker cadre with larger number of participants. The study was completed in a district with a non-governmental organization (NGO) partner who provided support to mentors in forms of transport, laptops, airtime et cetera that may not be possible for other resource-limited settings in and out of Malawi. Further BEC training and longitudinal mentorship with a larger cohort of providers from diverse geographic and care settings, and a control group with no mentorship will be critical to support and expand on the findings from this paper.

## Conclusion

Overall, the BEC course and subsequent longitudinal mentorship were feasible and acceptable to participants and mentors in the Malawian low resource context. The course content and concepts can be easily grasped and applied across all cadres providing emergency care services at the facility level. Follow-up longitudinal mentorship was highly acceptable and is likely important to cementing the course concepts for long-term retention of knowledge and skills. Further studies with larger cohorts in expanded settings, dedicated time and incentives for mentorship, and maintenance of trained staff to receive mentorship without competing priorities are needed.

## Supporting information

S1 File Pre-and post-course knowledge evaluation multiple choice questions. (DOCX)

S2 File BEC participant feedback for course and mentorship. (DOCX)
