# Correction: Basic emergency care course and longitudinal mentorship completed in a rural Neno District, Malawi: A feasibility, acceptability, and impact study

**DOI:** 10.1371/journal.pone.0310449

**Published:** 2024-09-10

**Authors:** The PLOS ONE Editors

## Notice of Republication

This article was republished on July 8, 2024, to address an issue identified post-publication. Please download this article again to view the correct version.

The article’s Data Availability statement is updated to: All relevant data are within the paper, its Supporting Information files, and via https://zenodo.org/records/13352658.
