# Supplementary material for: Basic emergency care course and longitudinal mentorship completed in a rural Neno District, Malawi: A feasibility, acceptability, and impact study
Source: PLoS One. 2023 Feb 6;18(2):e0280454. doi: 10.1371/journal.pone.0280454 (PMC9901771; doi:10.1371/journal.pone.0280454)
Supplement: S1 File — (DOCX) [file pone.0280454.s001.docx]

**Basic Emergency Care Course**

1. **Pre-Course Multiple Choice Questions**

**Name _______________________**

This brief and confidential multiple choice test has been created to help course facilitators understand your current level of knowledge in Emergency care. The grading of this exam will not contribute to your overall mark but, to receive the workbook and begin the course you must complete the test and give it to the local course coordinator. You will then receive the Basic Emergency Care Course Workbook.

Please note that there is **ONLY ONE** correct answer per question.

1. A 26-year-old man crashed his car into an electrical power pole and appears to be unconscious in the driver seat. The car is badly damaged and power lines have fallen onto the car which are still sparking. What is your first action?

A. Protect his cervical spine

B. Open his airway with a jaw thrust

C. Stay clear of the car until the power lines have been moved

D. Pull him from the car and lay him on the ground to begin your assessment

2. You are managing the airway of a 60-year-old man that has collapsed after having chest pain. You are worried about his airway and place an oropharyngeal airway (OPA) and he gags. What is your next step?

A. Replace the OPA with a smaller size

B. Remove the OPA and attempt to place a nasopharyngeal airway (NPA)

C. Leave the OPA in place and begin BVM ventilations

D. Remove the OPA and begin BVM ventilations

3. One of your staff members accidently ate peanuts with her lunch and she has a history of severe allergies. She is now having severe difficulty in breathing, swelling of the mouth and lips, and developing a rash on her face, chest, and arms. What is your next step?

A. Administer IM adrenaline

B. Administer salbutamol

C. Perform needle decompression of the chest

D. Start an IV

4. Which of the following is a sign of shock?

A. Capillary refill time of 1 second

B. Pulse rate of 90 beats per minute

C. Blood pressure of 130/70 mmHg

D. Skin that is cool, pale, and moist

5. A 24-year-old woman is complaining of difficulty in breathing for the past 3 days getting gradually worse. She has wheezing on exam and her vital signs are: blood pressure 140/80 mmHg, heart rate 110 beats per minute, respiratory rate 24 breaths per minute. What medication would you give her?

A. Naloxone

B. Salbutamol

C. Aspirin

D. Glucose

6. You are taking care of a 22-year-old man who has been sick for the past week. He is confused and has rapid, deep breathing and a sweet smell on his breath. His vital signs are: blood pressure is 90/50 mmHg and his heart rate is 120 beats per minute. His family states that he is a diabetic but has not been taking his insulin for the past few days because he was vomiting and could not eat. He has been urinating a lot. What is the most likely cause of his poor perfusion?

A. Diabetic Ketoacidosis

B. Spinal Cord Injury

C. Sepsis

D. Blood loss

7. A 32-year-old female collapsed and is brought to you for evaluation. She is awake now but feels dizzy. Her vital signs are: blood pressure is 84/48 mmHg, heart rate is 125 beats per minute, and respiratory rate is 18 breaths per minute. She is complaining of abdominal pain and a small amount of vaginal bleeding. What is the first action that you should take to manage her possible ectopic pregnancy?

A. Give salbutamol

B. Needle decompression

C. Intravenous fluids

D. Surgery

8. Which statement regarding shock in children is true?

A. Intravenous fluid resuscitation is very different for children with severe malnutrition

B. Normal vital sign ranges are the same for adult and children

C. A child’s blood pressure will drop earlier than an adult in shock

D. The capillary refill test is not useful in children

9. You are taking care of a 6 year-old that weighs 20kg and is not malnourished. He has had severe vomiting and diarrhoea for 3 days and now requires an intravenous fluid bolus. What is

the correct dose?

A. 500 mL bolus

B. 100 mL per hour

C. 200 mL bolus

D. 400 mL bolus

10. There is a cholera outbreak and you are taking care of a severely dehydrated 5-year-old child. The child is lethargic and hypotensive. What is your first priority?

A. Start an intravenous line and administer a fluid bolus

B. Listen to lung sounds

C. Wash your hands and wear gloves

D. Provide antibiotics

11. You are listening to the lungs of a person with difficulty in breathing and you hear a high pitched sound when breathing IN. What is this called?

A. Stridor

B. Wheezing

C. Crackles

D. Crepitations

12. You are taking care of a 40-year-old man with tuberculosis who has difficulty in breathing. On exam he has distended neck veins and muffled heart sounds. His vital signs are: blood pressure 80/40 mmHg, heart rate 130 beats per minute, respiratory rate 30 breaths per minute. What is the most likely cause of his difficulty in breathing?

A. Asthma

B. Pericardial tamponade

C. Pneumothorax

D. Flail chest

13. A 70-year-old woman comes in with severe chest pain. She has crackles in her lungs on both sides and distended neck veins. Her vital signs are blood pressure is 80/50 mmHg, and her capillary refill is 5 seconds. What is the most likely possible cause?

A. Severe allergy

B. Asthma

C. Severe infection

D. Heart attack

14. An ambulance brings you a 28-year-old man that was found unconscious at home. You do not see any trauma, but his both pupils are very small and he has a slow respiratory rate. What medication would you give?

A. Naloxone

B. Benzodiazepine

C. Adrenaline

D. Aspirin

15. Which of the following is a paediatric danger sign in the ABCDE assessment of a 6-month-old child?

A. Respiratory rate of 30 breaths per minute

B. Heart rate of 120 beats per minute

C. Grunts when breathing

D. Pink skin color

16. A 30-year-old male is found confused at the bus stop. He is brought to you for evaluation and is found to be responsive to verbal stimuli. His vital signs are: blood pressure 134/78 mmHg, heart rate 96 beats per minute, respiratory rate 16 breaths per minute. His pupils are equal and reactive and his blood glucose is 1.5 mmol/L. What is the recommended treatment?

A. Naloxone

B. Benzodiazepine

C. Glucose

D. Intravenous fluids

17. You are taking care of a 70-year-old man who suffered a stroke and is now complaining of a severe headache. He is very sleepy and confused. You are concerned for possible bleeding in the brain and are arranging transfer to a referral facility. What treatment can you provide while awaiting transfer?

A. Administer aspirin

B. Lay the patient flat at all times

C. Elevate the head of the bed to 30 degrees

D. Administer antibiotics

18. A 4-year-old boy is brought in by his mother for evaluation. She states he has been very confused and vomiting since this morning when he was playing near cleaning supplies. There has been no trauma or recent fevers. The vital signs are: blood pressure 100/50 mmHg, heart rate 106 beats per minute, respiratory rate 20 breaths per minute. What is the most likely cause of the new confusion and vomiting?

A. Psychiatric

B. Pneumonia

C. Sepsis

D. Ingestion

19. What do you need to worry about after you have given naloxone for an opioid overdose?

A. The naloxone may wear off before the opiate

B. The opioid may wear off before the naloxone

C. They may vomit from the naloxone

D. They may use an opioid again in the future

20. You are called outside to evaluate a 19-year-old male who fell and is now laying on the ground. He is confused but awake. He has a laceration on his head from the fall. Which of the following should you do first?

A. Arrange transfer for a CT scan

B. Control bleeding from the laceration

C. Check his blood glucose

D. Immobilize his cervical spine

21. A 3-year-old child has been run over by a car. The mother runs in to your clinic and gives you the child to look after. The child is unconscious and blue. The first thing you assess is:

A. Circulation

B. Airway while providing cervical spine immobilization

C. Look for head injury

D. Breathing

22. A 27-year-old man comes to the hospital with his arm wrapped in a shirt that is soaked in blood. He accidentally cut his arm on a large share of glass. You remove the shirt and there is a large amount of bleeding. What is your first step to control the bleeding?

A. Apply direct pressure

B. Begin a blood transfusion

C. Transfer rapidly to a referral facility

D. Apply a tourniquet

23. A 3-year-old child was burned by hot water. On her exam, you notice blistering on her arms that is very painful. You press two fingers gently onto the burn to assess the depth of burn. The burn turns white when you press it then the red color returns. What type of burn is this?

A. Chemical burn

B. Second degree (partial thickness)

C. Third degree (full thickness)

D. Electrical burn

24. A woman has been hit by a car. She is 30-weeks pregnant and the trauma primary and secondary surveys have been completed. She does not have a head injury but remains in cervical spine immobilization. She needs to be transported to the nearest obstetric centre. Which position is the would you transport her in?

A. Elevate head of bed to 30 degrees with cervical spine immobilization

B. Placed in the left lateral position with cervical spine immobilization

C. Asked to sit upright

D. Placed lying on her back

25. You are taking care of a 46-year-old man that was struck in the head with a hammer. He is bleeding from the head. When you speak to him, he does not open his eyes. When you ask him a question, there is no response. When you rub hard on his chest, he makes a sound. What is his AVPU score?

A. A

B. V

C. P

D. U

1. **Post-Course Multiple Choice Questions**

**Name: ____________________________**

This exam is designed to test your knowledge and retention of concepts taught within the Basic Emergency Care Course. Please **do not** use your course workbook or other resources during this exam. Circle the correct answer. You have 60 minutes to complete the 25 questions.

1. One of your friends comes to find you at work because she developed sudden onset of severe difficulty in breathing, swelling of the mouth and lips, and an itchy rash on her face, chest, and arms. What is your next step?

a. Administer Salbutamol

b. Perform needle decompression of the chest

c. Administer IM Adrenaline

d. Start an IV line

2. You are taking care of an 18-year-old girl who has been feeling poorly for the past week. She is now confused and has rapid, deep breathing. Her blood pressure is 86/58 mmHg and her heart rate is 126 beats per minute. She has been urinating a lot. Her family reports that she has “sugar problems” and you smell a sweet smell on her breath. What is the most likely cause of her poor perfusion?

a. Spinal Cord Injury

b. Diabetic Ketoacidosis

c. Diarrhoea

d. Blood loss

3. You are taking care of a 4-year-old that weighs 15kg and is not malnourished. He has had severe vomiting and diarrhoea since yesterday. He is unable to keep fluids down and you would like to give him an intravenous fluid bolus. What is the correct dose?

a. 100 mL per hour

b. 200 mL bolus

c. 300 mL bolus

d. 400 mL bolus

4. You are taking care of a 20-year-old man presents after a car accident with difficulty in breathing. He has no tenderness to palpation of his chest wall and no crepitus, but he has swollen neck veins and muffled heart sounds. His lung sounds are equal on both sides and he has equal chest rise and fall. His vital signs are: blood pressure 80/40 mmHg, heart rate 130 beats per minute, respiratory rate 30 breaths per minute. What is the most likely cause of his difficulty in breathing?

a. Asthma

b. Pneumothorax

c. Flail chest

d. Pericardial tamponade

5. You are listening to the lungs of a person with difficulty in breathing and you hear a high pitched sound when breathing OUT. What is this called?

a. Stridor

b. Wheezing

c. Crackles

d. Rhonchi

6. A 70-year-old man comes in for severe chest pain for the past one day. There is no history of trauma. His blood pressure is 70/50 mmHg, and his capillary refill is 5 seconds. He has crackles in both lungs and distended neck veins. What is the most likely possible cause?

a. Severe allergy

b. Heart attack

c. Asthma

d. Severe infection

7. Why should you monitor a patient closely after you have given naloxone for an opioid overdose?

a. The opioid may wear off before the naloxone

b. They may vomit from the naloxone

c. The naloxone may wear off before the opioid

d. They may try to use an opioid again

8. You are managing the airway of a 42-year-old man that was struck in the face with a metal pole. You are worried about his airway and plan to place an airway device. What is your next step?

a. After measuring from the angle of the jaw to the base of the ear, carefully place a nasopharyngeal airway in the mouth

b. After measuring from the nostril to the angle of the jaw, carefully place a nasopharyngeal airway in the nose

c. If the patient has no gag reflex, place an oropharyngeal airway

d. Place an oropharyngeal airway only if the patient’s gag reflex is present.

9. Which is a sign of shock?

a. Skin that is warm and dry

b. Pulse rate of 90 beats per minute

c. Blood pressure of 130/70 mmHg

d. Capillary refill time of 4 seconds

10. An 8-month-old boy is brought in by his father for evaluation. He states that the child was fine this morning but, when he returned home from work, he found the child to be tired appearing and not be very responsive with repeated vomiting for the past 5 hours. There have been no recent fevers, but he noted bruises along the child’s arms and legs. The vital signs are: blood pressure 100/50 mmHg, heart rate 106 beats per minute, respiratory rate 20. What is the most likely cause of the new lethargy and vomiting?

a. Psychiatric problem

b. Head Injury

c. Sepsis

d. Ingestion

11. You are called outside to evaluate a 21-year-old male who was punched in the face, knocked unconscious, and is now laying on the ground. He is confused but awake. He has an obviously deformed nose, which has a small amount of blood oozing from his left nostril. Which of the following should you do first?

a. Arrange transfer for a CT scan

b. Reset the deformed nose to stop the bleeding

c. Immobilize his cervical spine

d. Check his blood glucose

12. A 32-year-old female collapsed and is brought to you for evaluation. She is awake now but feels dizzy. She was seen by a doctor earlier today and was told that she is pregnant, but the pregnancy “is in the wrong place”. Her blood pressure is 88/52 mmHg, heart rate is 130 beats per minute, and respiratory rate is 22 breaths per minute. She is complaining of abdominal pain and a small amount of vaginal bleeding. What is the first treatment you should give her?

a. Give salbutamol

b. Needle decompression

c. Surgery

d. Intravenous fluids

13. On a very busy day in your clinic, one of the other patients tells you that they found a young man unconscious near the toilet. No one knows who the man is, and you do not see any signs of trauma. The only things you note on exam are that his pupils are equal but very small and he has a slow respiratory rate. What medication would you give?

a. Benzodiazepine

b. Adrenaline

c. Naloxone

d. Aspirin

14. A 46-year-old male is found confused at the bus stop. He is sweating and mumbling incoherently, but has his eyes open and is looking around. His vital signs are: blood pressure 134/78 mmHg, heart rate 96 beats per minute, respiratory rate 16 breaths per minute. His pupils are equal and reactive and his blood glucose is 1.5 mmol/L. What is the recommended treatment?

a. Naloxone

b. Benzodiazepine

c. Intravenous fluids

d. Glucose

15. Which of the following is a danger sign in the ABCDE assessment of a 2-year-old?

a. Chest in-drawing

b. Heart rate of 120 beats per minute

c. Pink skin color

d. Respiratory rate of 30 breaths per minute

16. A woman who is 37 weeks pregnant has been hit by a car. She has been stabilized and does not have a head injury but remains in cervical spine immobilization. Bystanders have laid her flat on the ground to immobilize her spine. While she is awaiting transport to the nearest Obstetric centre, she tells you she does not feel well and, when you check her blood pressure, it is 78/50 mmHg. What is the first thing you should do?

a. Prepare for emergency delivery

b. Place her in the left lateral position with cervical spine immobilization

c. Sit her upright

d. Advise she is in shock and needs to stay lying on her back

17. Which statement regarding shock in children is true?

a. Children with severe malnutrition should be given more intravenous fluids more rapidly if they are in shock.

b. Normal vital sign ranges are the same for adult and children

c. A child who is in shock may be able to maintain a normal blood pressure longer than an adult in shock

d. The capillary refill test is not useful in children

18. A 42-year-old woman crashed her car into a telephone pole. Bystanders have pulled her out of the car and she appears to be unconscious. The engine of the car is on fire. What is your first action?

a. Check whether it is safe to approach the patient

b. Open the patient’s airway

c. Instruct the bystanders on how to put padding under her shoulders

d. Run to the patient to immobilize C-spine

19. You are taking care of a 46-year-old man that was struck by a motorcycle and fell, striking his head on the road. He is bleeding from the head. When you speak to him there is no response. When you ask him a question there is no response. When you try to inflict pain by rubbing hard on his sternum there is no motor response. What is his GCS score?

a. 0

b. 3

c. 8

d. 15

20. A 26-year-old woman was knocked into the cooking fire by her husband. On her exam, you notice a white firm area on her thigh with burnt clothing stuck to it. You press two fingers gently onto the burn to assess the depth of burn. The burn does not change color when you press. What type of burn is this?

a. First degree (superficial)

b. Second degree (partial thickness)

c. Third degree (full thickness)

d. Fourth degree (deep full thickness)

21. An 18-year-old man presents after being cut with a machete. He has a tight dressing wrapped around his cut arm that is soaked in blood. As you remove the dressing the wound starts to bleed a lot. What is your first step to control the bleeding?

a. Begin a blood transfusion

b. Transfer rapidly to a referral facility

c. Apply direct pressure

d. Apply a tourniquet

22. You are taking care of a 20-year-old man who complained of a very severe headache and is now very sleepy and confused. You are concerned for possible bleeding in the brain and are arranging transfer to a referral facility. Which of these will be the most helpful while awaiting transfer?

a. Administer aspirin

b. Elevate the head of the bed to 30 degrees

c. Lay the patient flat at all times

d. Administer antibiotics

23. A 5-year-old child is brought into clinic by her mother for cough and difficulty breathing. She has chest in-drawing and wheezing on exam and her vital signs are as follows: blood pressure 110/80 mmHg, heart rate 120 beats per minute, respiratory rate 28 breaths per minute. What medication would you give her?

a. Naloxone

b. Salbutamol

c. Aspirin

d. Glucose

24. A 5-year-old girl fell from a tree. The mother runs in and gives you the child to look after. The child is unconscious and blue. The first thing you assess is:

a. The appropriate location to perform a needle decompression

b. Whether the breathing is adequate

c. Signs of allergic reaction

d. Airway with cervical spine immobilization

25. She states that the child has had a lot of thin, rice-water diarrhea over the past 24 hours and she is worried about cholera. What do you do first?

a. Start an intravenous line and administer a fluid bolus

b. Wash your hands and wear gloves

c. Listen to lung sounds

d. Provide antibiotics
