# Supplementary material for: Basic emergency care course and longitudinal mentorship completed in a rural Neno District, Malawi: A feasibility, acceptability, and impact study
Source: PLoS One. 2023 Feb 6;18(2):e0280454. doi: 10.1371/journal.pone.0280454 (PMC9901771; doi:10.1371/journal.pone.0280454)
Supplement: S2 File — (DOCX) [file pone.0280454.s002.docx]

1. **BEC PARTICIPANT COURSE FEEDBACK – IMMEDIATE**

Name: ________________________________________________________

What is your job title? _____________________________________________

Where do you work? *(please circle)*

Primary Health Centre First-level Hospital Referral/Specialized Hospital Other: ______

How long since you finished your training?

How long have you worked at your current job?

**We would like to know you what you thought about this course; what parts were good and what parts need to be changed.**

1. Regarding the course as a whole, what did you think of level of the content of the modules?

Very Somewhat Somewhat Inappropriate

Appropriate Appropriate Inappropriate

1. Regarding the course as a whole, how appropriate do you feel the content of the modules were to your setting?

Very Somewhat Somewhat Inappropriate

Appropriate Appropriate Inappropriate

1. Please let us know what you thought of the workbook for this course.
   1. Did you feel that the clarity and language of the workbook text was?

Very clear Somewhat clear Somewhat unclear Unclear

- 1. What did you think about the overall quality of the workbook?

Excellent Good Poor Unacceptable

1. Please let us know what you thought of the lectures for this course.
   1. Did you feel that the clarity and language of the text on the slides was?

Very clear Somewhat clear Somewhat unclear Unclear

- 1. What did you think about the overall quality of the lectures?

Excellent Good Poor Unacceptable

1. Please let us know what you thought about the Quick Cards for this course.
   1. Did you feel that the clarity and language of the Quick Card text was?

Very clear Somewhat clear Somewhat unclear Unclear

- 1. What did you think about the overall quality of the Quick Cards?

Excellent Good Poor Unacceptable

1. Please let us know what you thought about the case scenarios for this course.
   1. Did you feel that the clarity and language of the case scenario text was?

Very clear Somewhat clear Somewhat unclear Unclear

- 1. What did you think about the overall quality of the case scenarios?

Excellent Good Poor Unacceptable

1. Please let us know what you thought about the multiple choice questions for this course.
   1. Did you feel that the clarity and language of the multiple choice text was?

Very clear Somewhat clear Somewhat unclear Unclear

- 1. What did you think about the overall quality of the multiple choice questions?

Excellent Good Poor Unacceptable

1. I believe that the BEC course was a productive use of my time.

Strongly Disagree Disagree Neutral Agree Strongly Agree

1. I would recommend the BEC course to other healthcare providers

Strongly Disagree Disagree Neutral Agree Strongly Agree

1. How effective do you feel your instructors were in teaching this course?

Excellent Good Poor Unacceptable

Please explain:

1. What specific things did you like about this course?
2. What would you change about this course?
3. **BEC PARTICIPANT COURSE AND MENTORSHIP FEEDBACK – 1 YEAR POST-COURSE**

Please answer the questions below regarding your participating in the BEC course and follow up mentorship program. Answers will be kept anonymous.

1. Do you feel your participation in the BEC course helped you improve your knowledge and skills to care for emergency patients? If so, how?
2. Did having a BEC mentor available after the training improve your understanding and retention of the concepts with further learning? If so, how? If not, please explain why you felt the mentorship was not needed.
3. In what ways could the training or mentorship program have been improved to help you better care for emergency patients?
4. Are there any additional suggestions or comments you have regarding your participation in the BEC course and mentorship program?
